# Effect of variation of average pore size and specific surface area of ZnO electrode (WE) on efficiency of dye-sensitized solar cells

**DOI:** 10.1186/1556-276X-9-575

**Published:** 2014-10-14

**Authors:** Nitin A Jadhav, Pramod K Singh, Hee Woo Rhee, Bhaskar Bhattacharya

**Affiliations:** 1 Materials Research Laboratory, School of Basic Sciences and Research, Sharda University, Greater Noida 201310, India; 2 Post-graduate Department of Chemistry, Tuljaram Chaturchand College, Baramati, Pune 413102, India; 3 Institute for Microsystems Technology, Vestfold University College, Tonsberg 3103, Norway; 4 Department of Chemical and Biomolecular Engineering, Sogang University, Seoul, South Korea

**Keywords:** Mesoporous ZnO, EISA, Copolymer, DSSC

## Abstract

Mesoporous ZnO nanoparticles have been synthesized with tremendous increase in specific surface area of up to 578 m^2^/g which was 5.54 m^2^/g in previous reports (J. Phys. Chem. C 113:14676-14680, 2009). Different mesoporous ZnO nanoparticles with average pore sizes ranging from 7.22 to 13.43 nm and specific surface area ranging from 50.41 to 578 m^2^/g were prepared through the sol-gel method via a simple evaporation-induced self-assembly process. The hydrolysis rate of zinc acetate was varied using different concentrations of sodium hydroxide. Morphology, crystallinity, porosity, and *J*-*V* characteristics of the materials have been studied using transmission electron microscopy (TEM), X-ray diffraction (XRD), BET nitrogen adsorption/desorption, and Keithley instruments.

## Background

Zinc oxide is one of the principal multifunctional materials due to its unique characteristics having applications in catalysis [1,2], actuators and sensors [3,4], drug delivery [5], and optoelectronics [6,7]. ZnO nanostructures varying from nanoparticles, nanorods, and nanotriangles to nanoribbons [8-10] have already been developed. The particle size in nano-range shows tremendous increase in specific surface area with low dimensions which results in a wider range and efficient application in a variety of fields.

For efficient device applications, nanomaterials having a large specific surface area are the prime need of the present scenario, and nanomaterials with porous morphology are an additional important feature for device application. Very recently, nanostructures with a highly ordered porous network and having a large specific surface area with an optimum pore size such as TiO_2_[11-13], ZnO [14-16], SiO_2_[17], and SnO_2_[18] have been developed, and most crystalline mesoporous metal oxides such as Co_3_O_4_, NiO, and MnO_2_[19,20] were synthesized using a hard template like Al_2_O_3_ mesoporous silica, mesoporous carbon [21], SBA-15, and KIT-6, which require high-temperature synthesis. Recently, reports on the synthesis of mesoporous metal oxides such as the highly ordered network of porous TiO_2_ and SiO_2_ using soft templates like block copolymers have also been found, which need low-temperature synthesis. Synthesis of ZnO structures also available in the literature. Bhattacharyya and Gedanken have reported the synthesis of mesoporous ZnO and Ag-ZnO nanocomposites by microwave-assisted and sonochemical routes. Gao and Wang [22] have also recently reported the synthesis of cage- and shell-like porous ZnO structures grown by self-assembly of ZnO nanocrystals. Very recently, Pal et al. have reported the ultrasound-assisted synthesis of mesoporous ZnO nanostructures of different porosities [14].

Nowadays, dye-sensitized solar cell (DSSC) technology based on the ZnO photoelectrode has been explored extensively. Due to the advanced photocatalytic property of ZnO, some efforts are also found on the fabrication of a DSSC using ZnO as the working electrode. ZnO is a wide-band gap semiconductor that possesses an energy band structure and physical properties similar to those of TiO_2_ but has higher electronic mobility that would be favorable for fast electron transport, with reduced recombination loss when used in DSSCs. Many studies have already reported on the use of a ZnO material for application in DSSCs. Although the conversion efficiencies of ZnO are much lower than those of TiO_2_, ZnO is still a distinguished alternative to TiO_2_ due to its ease of crystallization and anisotropic growth. These properties allow ZnO to be produced in a wide variety of nanostructures, thus possessing unique properties for electronics, optics, or photocatalysis [23]. Recent studies on ZnO nanostructure-based DSSCs have delivered many new concepts, leading to a better understanding of photoelectrochemically based energy conversion. This, in turn, would speed up the development of DSSCs that are associated with TiO_2_. One of the defining features of nanostructures is their size in the nanometer scale. This, first of all, provides the nanostructures with a large specific surface area. It may also result in many particular behaviors in electron transport or light propagation in view of the surface effect. Those nanostructural forms of ZnO which were developed during the past several decades mainly include nanowires (or nanorods), nanotubes, nanobelts, nanosheets, nanotips, and nanoparticles [24].

In the present article, we report on crystalline mesoporous ZnO with the highest surface area of up to 578 m^2^/g as compared with previous reports, with variation of pore size ranging from 7.22 to 13.43 nm. We have shown that photoelectrode films with nanostructured ZnO can significantly enhance solar cell performance by an increase in average pore size for direct transport pathways of photo-excited electrons and with efficient scattering centers for enhanced light-harvesting efficiency. It is shown that specific surface area plays an important role in increasing solar cell performance, but pore size is also a significant factor which affects the cell parameter; also, the size of the nanocrystallite can alter the electrode-electrolyte junction which affects the open-circuit voltage *V*_oc_ of DSSCs. In the present work, we have used a solid polymer electrolyte, since an liquid electrolyte has much more limitations like evaporation leakage and decrease in efficiency, but for the solid polymer electrolyte, it is very significant as the efficiency remains constant for more than a year.

## Methods

### Materials

Zinc acetate (Thermo Fisher Scientific, Waltham, MA, USA), Pluronic F-127 (extra pure, Sigma-Aldrich, St. Louis, MO, USA), methyl alcohol, (CH_2_OH; Rankem Chemicals, Faridabad, India), and sodium hydroxide (NaOH; Thermo Fisher Scientific, Waltham, MA, USA) were used as received without further purification.

### Synthesis

The synthesis of mesoporous ZnO was carried out using the triblock copolymer-templated sol-gel method via an evaporation-induced self-assembly (EISA) process. Zn sol can be prepared by adding 0.38 M of zinc acetate to 15 ml of already-prepared 0.001 M F-127 copolymer template methanolic solution under vigorous magnetic stirring for 3 h. NaOH solutions were prepared by adding different amounts of NaOH in 5 ml of methanol under vigorous magnetic stirring for 3 h. The amount of the NaOH was varied in the final mixture solution as 0.15, 0.2, 0.25, 0.3, 0.35, 0.4, 0.45, and 0.5 M. Then, it was dropwise added to the previously prepared template zinc acetate solution under magnetic stirring. The stirring process was continued for 12 h until the mixture initially forms a colorless sol and then white gel.

### Fabrication of a DSSC

The resulting paste was then used to fabricate the working electrode using the well-known doctor blade method as reported in [25]. The synthesized paste was employed on a cleaned and blocking layer-coated fluorine-doped tin oxide (FTO) glass and calcinated at 315°C for 12 h at a heating rate of 1°C/min. The calcinated working electrode was then soaked in (0.5 mM in ethanol) dye solution overnight and washed with ethanol to remove excess of the dye adsorbed on the surface of the electrode. The solid polymer electrolyte was prepared by dissolving the polyethylene oxide (PEO)/PEG blend in acetonitrile and adding NaI and I_2_ (75:25 *w*/*w*%) and iodine (10 *w*/*w*%), respectively. The platinum counter electrode was prepared by spin coating the H_2_PtCl_6_ solution on a clean FTO glass and heated at 400°C for 30 min. A drop of the above prepared electrolyte was sandwiched between the fabricated working electrode and counter electrode and used to record the current density-voltage (*J*-*V*) curve using a Keithley 2400 sourcemeter (Keithley Instruments Inc., Cleveland, OH, USA).

### Characterizations

The structure, texture, and morphology of the synthesized samples were examined using transmission electron microscopes (TEM; models JEM-2010 and JEOL JEM 2100 F, JEOL Ltd., Akishima, Tokyo, Japan) operating at 200 kV. For the TEM analysis, a small amount of the sample was dispersed in 95% ethanol. For the structural determination, powder X-ray diffraction (XRD) patterns were recorded with a Rigaku X-ray diffractometer (Rigaku Corporation, Tokyo, Japan) using CuKα radiation (*λ* =1.5405 Å). Surface area, pore volume, pore size distribution, and pore diameter were measured by a BET Quantachrome Autosorb AS1WIN instrument (Quantachrome Instruments, Boynton Beach, FL, USA). To analyze the pore size, the sample is outgassed at 300°C for 7 h. The DSSC parameters like open-circuit voltage (*V*_oc_), short-circuit current density (*J*_sc_),fill factor (FF), and conversion efficiency (*η*) were measured by a Keithley sourcemeter (model no. 4200, Keithley Instruments Inc., Cleveland, OH, USA).

## Results and discussion

**Figure 1:**
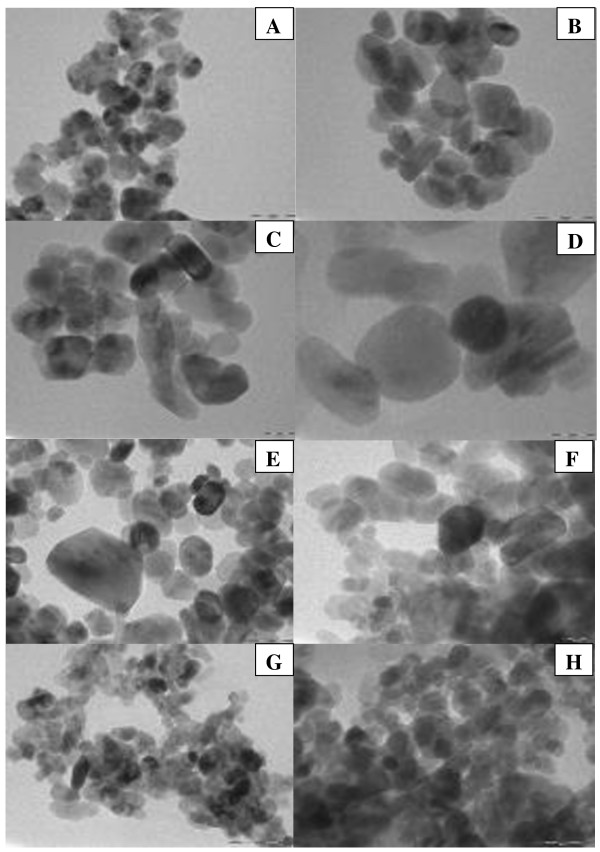
**Typical TEM images of the mesoporous ZnO samples synthesized using different concentrations of NaOH. (A)** 0.15 M, **(B)** 0.2 M, **(C)** 0.25 M, **(D)** 0.3 M, **(E)** 0.35 M, **(F)** 0.4 M, **(G)** 0.45 M, and **(H)** 0.5 M. The samples were calcinated at 315°C for 12 h.

**Figure 2:**
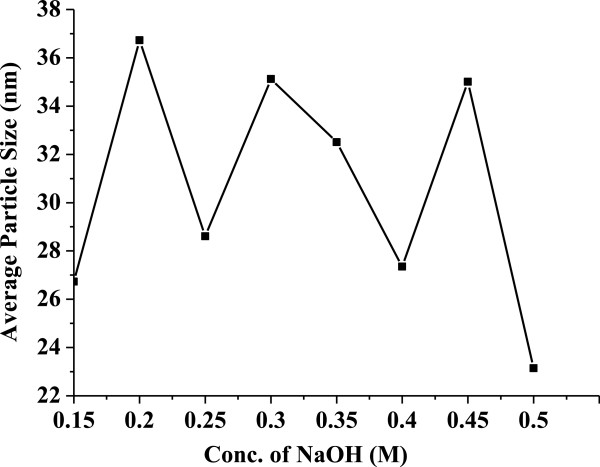
**Variation of average particle size versus the mesoporous ZnO samples synthesized using different concentrations of NaOH.** The concentrations used were 0.15, 0.2, 0.25, 0.3, 0.35, 0.4, 0.45, and 0.5 M. The samples were calcinated at 315°C for 12 h.

**Figure 3:**
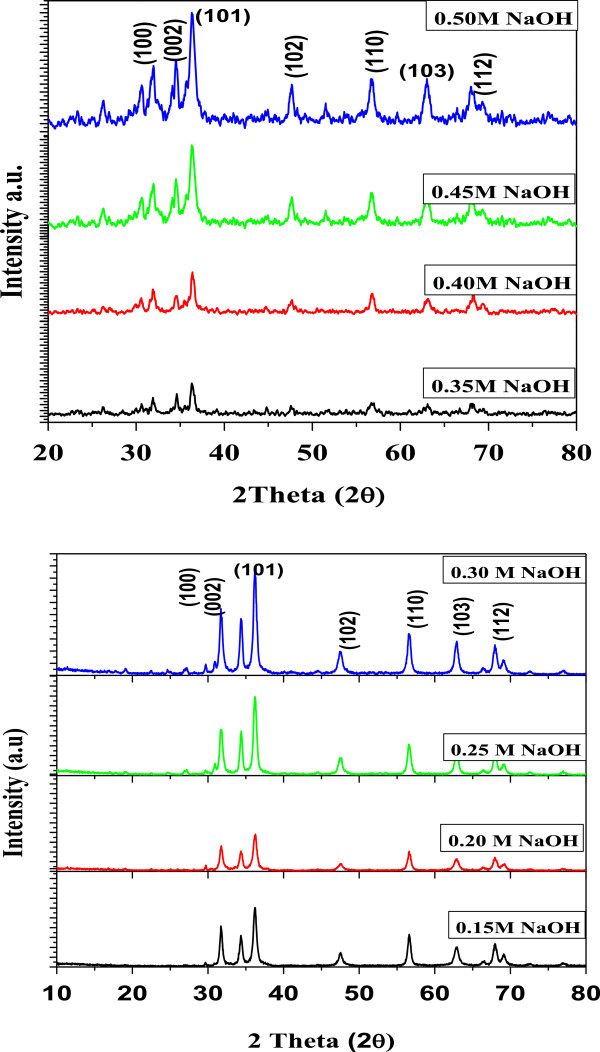
**XRD spectra of the mesoporous ZnO samples synthesized using different concentrations of NaOH.** The concentrations used were 0.15, 0.2, 0.25, 0.3, 0.35, 0.4, 0.45, and 0.5 M. The samples were calcinated at 315°C for 12 h. The diffraction peaks are identified using the standard JCPDS data (card no. 01-071-6424).

Figure 1 shows the typical TEM images of the mesoporous ZnO samples synthesized using different concentrations of NaOH: (A) 0.15 M, (B) 0.2 M, (C) 0.25 M, (D) 0.3 M, (E) 0.35 M, (F) 0.4 M, (G) 0.45 M, and (H) 0.5 M. The samples were calcinated at 315°C for 12 h. Particles with rod shape or cylindrical shape were obtained which can be clearly seen from the TEM images. Change in shape could be due to the variation of NaOH concentration. The rate of nucleation and growth depends upon the rate of hydrolysis by NaOH. We know that the rate of nucleation and growth is the deciding factor for the formation of different shapes and geometries of the nanoparticles. It also plays a very important role on the average particle size, average pore size, and also on specific surface area which is discussed later. It is well known that as concentration of NaOH varies from lower concentration to higher concentration, the hydrolysis varies and the rate of nucleation and growth also varies, resulting in the formation of particles with varied geometries. Due to this variation, particles with random size are formed. The TEM images show the average particle size ranging from 23 to 36 nm. Variations of the average particle size of ZnO samples synthesized using different concentrations of NaOH have been presented in Figure 2 as obtained from TEM.The wide-angle XRD patterns (Figure 3) for all synthesized samples revealed good crystallinity of zincite form with perfect hexagonal geometry which is confirmed by exactly matching with standard JCPDS data (card no. 01-071-6424). The zincite for of ZnO is rarely available in nature and mostly used in semiconductor technology.

**Figure 4:**
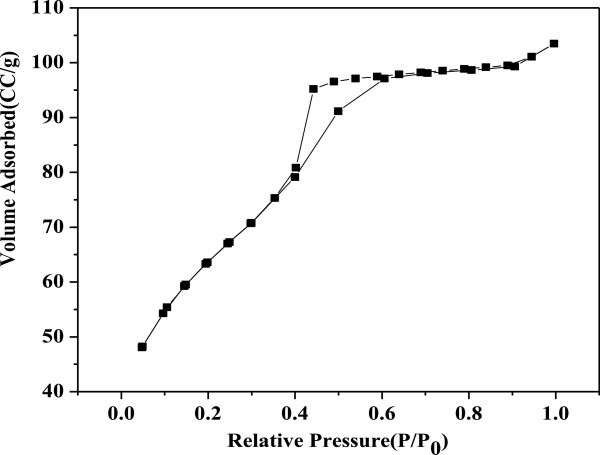
**Representative nitrogen adsorption/desorption isotherms for the mesoporous ZnO samples synthesized using different concentrations of NaOH.** The concentrations used were 0.15, 0.2, 0.25, 0.3, 0.35, 0.4, 0.45, and 0.5 M. The samples were calcinated at 315°C for 12 h.

Nitrogen adsorption/desorption isotherms for the mesoporous ZnO samples synthesized at 0.15 M NaOH are given in Figure 4. Similar isotherms were obtained for all other samples synthesized at different NaOH concentrations. It may be noted that all of these samples were calcined at 315°C for 12 h. As can be noticed, all the samples revealed similar and type-IV isotherms (IUPAC classification) which are associated to a mesoporous structure of better pore size uniformity. The sharp desorption at about *P*/*P*_0_ = 0.45 indicates sudden evaporation of the adsorbate from cylindrical-shaped pores.

**Figure 5:**
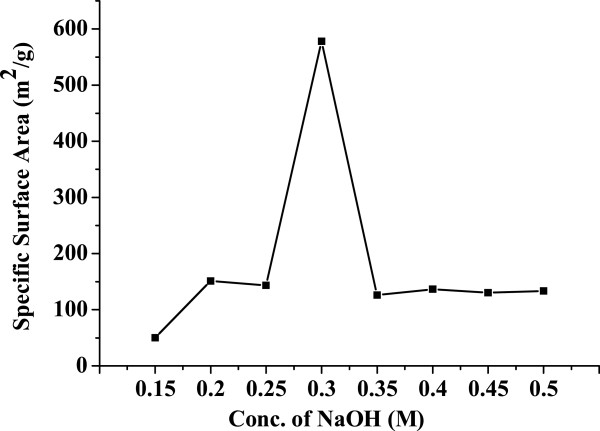
**Variation of specific surface area versus the mesoporous ZnO samples synthesized using different concentrations of NaOH.** The concentrations used were 0.15, 0.2, 0.25, 0.3, 0.35, 0.4, 0.45, and 0.5 M. The samples were calcinated at 315°C for 12 h.

**Figure 6:**
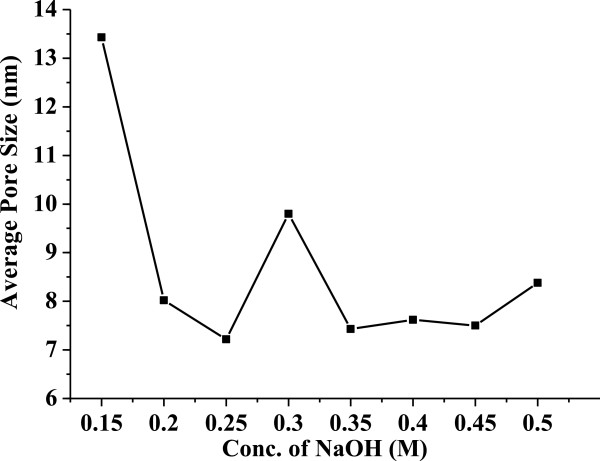
**Variation of average pore size versus the mesoporous ZnO samples synthesized using different concentrations of NaOH.** The concentrations used were 0.15, 0.2, 0.25, 0.3, 0.35, 0.4, 0.45, and 0.5 M. The samples were calcinated at 315°C for 12 h.

Figure 5 shows the variation of specific surface area versus the mesoporous ZnO samples synthesized using different concentrations of NaOH: 0.15, 0.2, 0.25, 0.3, 0.35, 0.4, 0.45, and 0.5 M. All the samples were calcinated at 315°C for 12 h. From the figure, it is observed that the specific surface area initially increases with an increase in NaOH concentration. The surface area suddenly increases at optimum NaOH concentration, and then it decreases with increasing NaOH concentration. It is because the rate of nucleation and growth of the mesoporous material which takes place during the reaction is the deciding factor for controlling the surface area pore size and also particle size. The rate of nucleation and growth depends upon the rate of hydrolysis which depends upon the reaction parameters like pH, temperature, and concentration of the precursor and base. In the present case, as the concentration of the base NaOH increases, initially the rate of hydrolysis slowly increases which leads to the gradual increase in specific surface area. But this trend is observed up to a certain concentration, and at optimum concentration of the base, there is a sudden and tremendous increase in specific surface area. After this NaOH concentration, the rate of nucleation and growth becomes very fast which leads to disordered porous structures and the specific surface area decreases. However, the specific surface area remains constant with increasing NaOH concentration. In the present work, we could observe the highest specific surface of upto 578 m^2^/g. This is much higher than the other reports found to date [14].Figure 6 shows the variation of average pore size versus the mesoporous ZnO samples synthesized using different concentrations of NaOH: 0.15, 0.2, 0.25, 0.3, 0.35, 0.4, 0.45, and 0.5 M. The samples were calcinated at 315°C for 12 h. It was noticed that at lower base concentration, larger pore size formation takes place, but as the concentration of the base increases, there is a sudden decrease in pore size. This happens due to the increase in hydrolysis rate, and faster nucleation and growth of ZnO take place. As discussed earlier, as the concentration of NaOH increases, there is increase in hydrolysis rate which leads to increase in nucleation and growth rate. But in the case of the porous structure, the hydrolysis as well as nucleation and growth rate plays a very important role as the porous structure is very much sensitive to these parameters. As the hydrolysis rate as well as nucleation and growth rate increases, the porous structure collapses which leads to a decrease in pore size. Hence, with increase in NaOH concentration, there is a decrease in average pore size.

### Dye-sensitized solar cells

**Figure 7:**
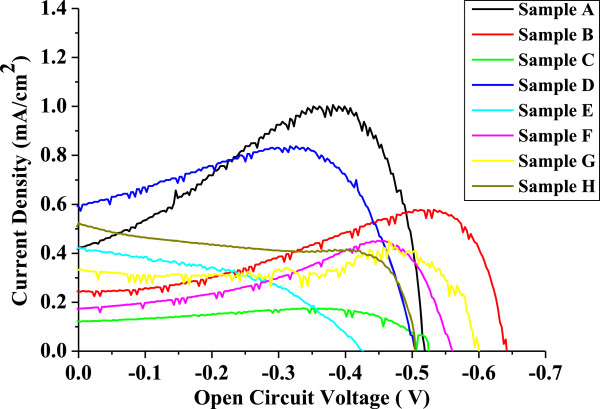
**Representative *J *- *V *curves for mesoporous ZnO samples synthesized using different concentrations of NaOH.** The concentrations used were 0.15 M (sample A), 0.2 M (sample B), 0.25 M (sample C), 0.3 M (sample D), 0.35 M (sample E), 0.4 M (sample F), 0.45 M (sample G), and 0.5 M (sample H). The samples were calcinated at 315°C for 12 h.

The synthesized ZnO samples from different concentrations of NaOH were used to fabricate the working electrode of DSSCs. The photocurrent *J*-*V* characteristics of the DSSCs fabricated with the different morphologies of ZnO photoanodes are shown in Figure 7, and all the parameters are summarized in Table 1.

**Table 1:** Comparison of specific surface area, average pore size, average particle size, and DSSC parameters

**Samples**	**Specific surface area (m**^**2**^**/g)**	**Average pore size (nm)**	**Average particle size (nm)**	***V***_**oc**_**(V)**	***J***_**sc**_**(mA)**	**Efficiency (%)**
A	50.41	13.43	26.72942	0.4874	1.00	0.2878
B	151.1	8.02	36.73	0.6223	5.78 × 10^−1^	0.3181
C	143.5	7.22	28.61	0.5103	1.75 × 10^−1^	0.1136
D	578	9.80	35.12	0.5553	8.36 × 10^−1^	0.2112
E	126	7.43	32.51	0.5478	4.21 × 10^−1^	0.03006
F	136.5	7.62	27.35084	0.5189	4.51 × 10^−1^	0.08848
G	130.2	7.50	35.01	0.6321	4.50 × 10^−1^	0.079
H	133.3	8.38	23.13805	0.4844	5.24 × 10^−1^	0.1047

*V*_oc_, *J*_sc_, and efficiencies are the DSSC parameters.

**Figure 8:**
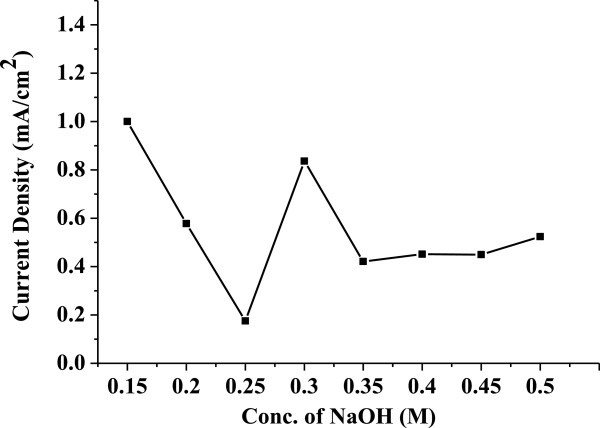
**Variation of short-circuit current density (*J***_**sc**_**) versus mesoporous ZnO samples synthesized using different concentrations of NaOH.** The concentrations used were 0.15, 0.2, 0.25, 0.3, 0.35, 0.4, 0.45, and 0.5 M. The samples were calcinated at 315°C for 12 h.

*J*_sc_ obtained from different cells is plotted against samples synthesized with different morphologies as shown in Figure 8, and it is observed that the obtained *J*_sc_ values follow the same trend followed by pore size (see the pore size plot in Figure 6), i.e., for the cells fabricated using the photo-electrode, which have higher pore size, the value of *J*_sc_ is high, and at lower pore size, the value of *J*_sc_ is low; as pore size decreases, *J*_sc_ also decreases. This is attributed to the radius of gyration of polymers used in the electrolyte (the PEO radius of gyration is 13.7 nm). As pore size increases more than the radius of gyration of the polymer, there are lot of chances for the polymer chain to penetrate into the pores of the electrode and exchange electrons with the dye molecule adsorbed on the surface of the electrode as well as the dye molecule inside the pore, which get reduced through the polymer electrolyte, hence resulting in the increase of photocurrent. As pore size decreases, it is difficult for the polymer chain to penetrate into the pore and exchange electrons with the dye molecule adsorbed inside the pores, resulting to a possible decrease of the photocurrent.

**Figure 9:**
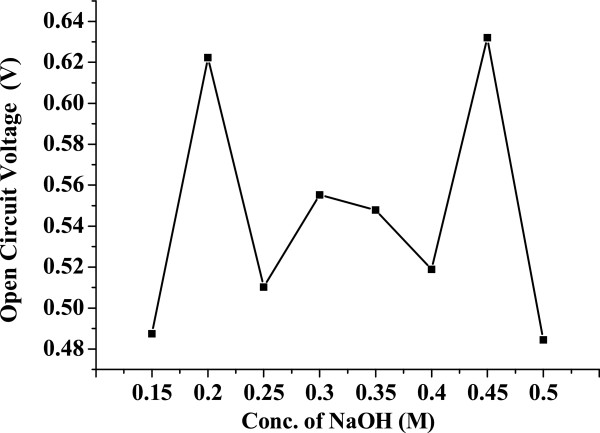
**Variation of open-circuit voltage (*V***_**oc**_**) versus the mesoporous ZnO samples synthesized using different concentrations of NaOH.** The concentrations used were 0.15, 0.2, 0.25, 0.3, 0.35, 0.4, 0.45, and 0.5 M. The samples were calcinated at 315°C for 12 h.

Figure 9 shows the variation of open-circuit voltage (*V*_oc_) for different ZnO samples synthesized as detailed above. It is observed that the *V*_oc_ follows the trend of particle size, i.e., as particle size increases, the *V*_oc_ increases and vice versa (see the particle size plot in Figure 2).

It is known that *V*_oc_ is the potential difference between the redox potential at the counter electrode and the conduction band of the semiconductor layer. It may be noted that at smaller sizes of the particle, the band structure does not prevail inside these particles. It is only the compact structure (cluster) of these particles which is taken as equivalent to the band structure. In such condition, smaller particles will result in incomplete band structure, and hence, the flat band potential will become lower [26]. Any increase in particle size will lead to better band formation, proper band bending, and higher flat band potential. Such change will not only affect on the *V*_oc_, but also lead to varied overlapping of the bands of ZnO and the excited level of the dye and hence a change in photocurrent. From all of the above discussion, we could identify that at 0.3 M NaOH concentration, the highest specific surface area of up to 578 m^2^/g is obtained, but the highest pore size of 13.43 nm is obtained at 0.15 M NaOH concentration. This shows that the highest *J*_sc_ is 1 mA.

**Figure 10:**
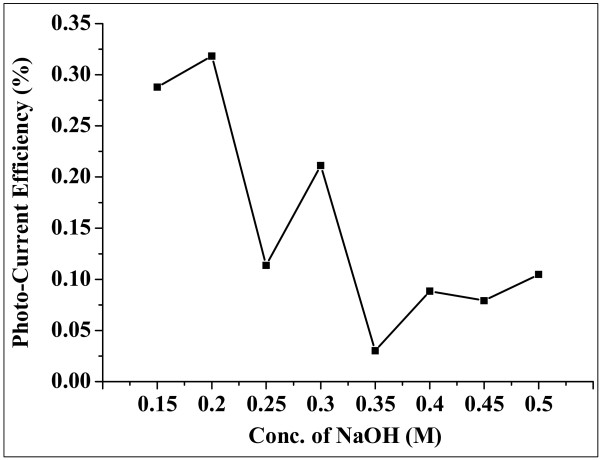
**Variation of photocurrent conversion efficiency (%) versus mesoporous ZnO samples synthesized using different concentrations of NaOH.** The concentrations used were 0.15, 0.2, 0.25, 0.3, 0.35, 0.4, 0.45, and 0.5 M. The samples were calcinated at 315°C for 12 h.

Changes in *V*_oc_ and *J*_sc_ are reflected on the photo-conversion efficiency of the DSSC. Variation in conversion efficiency for all synthesized samples has been shown in Figure 10 which is in accordance with the change in particle size and *V*_oc_ as discussed above.

## Conclusions

We present a synthesis strategy of preparing mesoporous ZnO powder in bulk amount through the triblock copolymer-templated sol-gel method via an EISA process. We have successfully modified the working electrode of DSSCs. Additionally, we have been able to produce the modified structure, texture, morphology, pore size, as well as surface area. Direct co-relation has been established between increase/decrease in pore size on the short-circuit current of DSSCs and increase or decrease in particle size on the open-circuit voltage of DSSCs. We have successfully synthesized mesoporous ZnO with the highest specific surface area of upto 578 m^2^/g available in the literature. Also, we have successfully achieved the highest short-circuit current density of up to 1 mA with 13.47-nm pore size. These findings open up new challenges for modifying the other components of DSSCs to improve the cell performance.

## Abbreviations

DSSC: dye-sensitized solar cell; *J*_sc_: short-circuit current density; *V*_oc_: open-circuit voltage; WE: working electrode; XRD: X-ray diffraction.

## Competing interests

The authors declare that they have no competing interests.

## Authors' contributions

NAJ carried out all the synthesis and DSSC fabrication work. HWR contributed to this work by helping in the characterization through TEM and BET. PKS and BB participated in the sequence alignment, helped in the discussion and in predicting the conclusion of the obtained results, and drafted the whole manuscript. All authors read and approved the final manuscript.
